# Immunoprotection Strategies in β‐Cell Replacement Therapy: A Closer Look at Porcine Islet Xenotransplantation

**DOI:** 10.1002/advs.202401385

**Published:** 2024-06-17

**Authors:** Sarah Grimus, Victoria Sarangova, Petra B. Welzel, Barbara Ludwig, Jochen Seissler, Elisabeth Kemter, Eckhard Wolf, Asghar Ali

**Affiliations:** ^1^ Chair for Molecular Animal Breeding and Biotechnology Gene Center and Department of Veterinary Sciences LMU Munich D‐81377 Munich Germany; ^2^ Center for Innovative Medical Models (CiMM) LMU Munich D‐85764 Oberschleißheim Germany; ^3^ Interfaculty Center for Endocrine and Cardiovascular Disease Network Modelling and Clinical Transfer (ICONLMU) LMU Munich D‐81377 Munich Germany; ^4^ Leibniz‐Institut für Polymerforschung Dresden e.V. Max Bergmann Center of Biomaterials Dresden D‐01069 Dresden Germany; ^5^ Department of Medicine III University Hospital Carl Gustav Carus Technische Universität Dresden D‐01307 Dresden Germany; ^6^ Paul Langerhans Institute Dresden of the Helmholtz Center Munich at the University Hospital Carl Gustav Carus and Faculty of Medicine of the Technische Universität Dresden D‐01307 Dresden Germany; ^7^ German Center for Diabetes Research (DZD e.V.) D‐85764 Neuherberg Germany; ^8^ DFG‐Center for Regenerative Therapies Dresden Technische Universität Dresden D‐01307 Dresden Germany; ^9^ Medizinische Klinik und Poliklinik IV Diabetes Zentrum – Campus Innenstadt Klinikum der Ludwig‐Maximilians‐Universität München D‐80336 Munich Germany

**Keywords:** encapsulation, genetic engineering, immunomodulation, pancreatic islets, pig, xenotransplantation

## Abstract

Type 1 diabetes mellitus (T1DM) is characterized by absolute insulin deficiency primarily due to autoimmune destruction of pancreatic β‐cells. The prevailing treatment for T1DM involves daily subcutaneous insulin injections, but a substantial proportion of patients face challenges such as severe hypoglycemic episodes and poorly controlled hyperglycemia. For T1DM patients, a more effective therapeutic option involves the replacement of β‐cells through allogeneic transplantation of either the entire pancreas or isolated pancreatic islets. Unfortunately, the scarcity of transplantable human organs has led to a growing list of patients waiting for an islet transplant. One potential alternative is xenotransplantation of porcine pancreatic islets. However, due to inter‐species molecular incompatibilities, porcine tissues trigger a robust immune response in humans, leading to xenograft rejection. Several promising strategies aim to overcome this challenge and enhance the long‐term survival and functionality of xenogeneic islet grafts. These strategies include the use of islets derived from genetically modified pigs, immunoisolation of islets by encapsulation in biocompatible materials, and the creation of an immunomodulatory microenvironment by co‐transplanting islets with accessory cells or utilizing immunomodulatory biomaterials. This review concentrates on delineating the primary obstacles in islet xenotransplantation and elucidates the fundamental principles and recent breakthroughs aimed at addressing these challenges.

## Graphical abstract

Type 1 diabetes mellitus (T1DM) arises from insulin deficiency resulting from the destruction of pancreatic β‐cells. Treatment usually entails daily insulin injections, but challenges endure. Allogeneic pancreatic islet transplantation holds promise, yet its viability is constrained by organ scarcity. Xenotransplantation of porcine islets provides an alternative, impeded by immune rejection. Strategies such as genetic modification and immunoisolation aim to improve graft survival, explored in this review (created with Biorender.com).

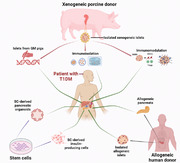

## 1 The Necessity of Islet Xenotransplantation and Associated Challenges

Diabetes mellitus (DM) is one of the most prevalent medical conditions demanding improved medical treatment. The global diabetic population surpasses 500 million, with projections indicating a surge to 695 million by 2045.^[^ ^1^ ^]^ Along with disrupted pancreatic endocrine function, diabetes can lead to severe complications such as nephropathy, cardiovascular diseases, retinopathy, and neuropathy.^[^ ^1^, ^2^ ^]^ Two vastly agreed pathophysiologies of diabetes include pancreatic β‐cell destruction in T1DM and insulin resistance coupled with insulin secretory defects in type 2 DM (T2DM).^[^ ^3^ ^]^ T1DM affects 5%–10% of the total diabetic population,^[^ ^1b^ ^]^ with an anticipated increase in incidence.^[^ ^4^ ^]^ While insulin therapy is currently a life‐saving intervention for T1DM, it cannot replicate crucial functional aspects of pancreatic β‐cells. Notably, it lacks emulation of the physiological kinetics of insulin release in response to glucose, the first‐pass hepatic insulin extraction,^[^ ^1^, ^5^ ^]^ and the crosstalk between β‐ and α‐cells within pancreatic islets, which is crucial for metabolic regulation.^[^ ^5^ ^]^

The development of rapidly acting insulin analogs, novel insulin pump technologies, and continuous glucose sensors (CGM) equipped with highly sophisticated algorithms, operating in the interstitial fluid (semi‐closed‐loop artificial pancreas), have significantly improved the quality of clinical care of diabetes. These advancements have increased time in range (TIR) values, the percentage of time a patient spends within the target glucose ranges. Despite this improvement, comprehensive observational data of high quality is still limited. Challenges persist for individuals with T1DM in reaching treatment objectives and averting complications. In the context of current hybrid closed‐loop systems, the users are still tasked with controlling the continuity of the subcutaneous catheters, validating CGM blood glucose data in case of potential sensor inaccuracies, calculating the carbohydrate intake, and providing input regarding planned physical activities.^[^ ^6^ ^]^ Psychological aspects, such as reluctance to have the closed‐loop system consistently connected to the body or disruptions to daily life due to frequent alarm messages, also remain significant barriers.^[^ ^7^ ^]^ Achieving a fully closed‐loop system that eliminates the need for user input, or the development of fully implanted devices, is a distant reality.^[^ ^6c^ ^]^

Notably, 25% of T1DM patients experience hypoglycemia unawareness, and the use of insulin therapy may lead to severe hypoglycemia, resulting in a hypoglycemic coma.^[^ ^8^ ^]^ For such patients, β‐cell replacement therapy emerges as a superior therapeutic option.^[^ ^5^ ^]^ Recent data suggests that globally, up to 5 million diabetes patients could benefit from β‐cell replacement therapy.^[^ ^2d^ ^]^ Allotransplantation of the whole pancreas or isolated pancreatic islets is a potential treatment option for T1DM; the former is less commonly performed due to its highly invasive nature^[^ ^9^ ^]^ and the requirement for lifelong immunosuppression.^[^ ^10^ ^]^ Allogeneic islet transplantation into the portal vein using the Edmonton protocol is the most prevalent clinical approach for β‐cell replacement therapy.^[^ ^1b^ ^]^ A significant loss of islets occurs during/after transplantation into the portal vein and often necessitates the use of more than one pancreas to obtain sufficient islet mass for a single recipient.^[^ ^9^ ^]^ The limited availability of suitable human donors also poses a substantial challenge to allotransplantation. A promising alternative lies in the use of insulin‐producing β‐cells derived from human embryonic stem cells (hESC) or other human pluripotent stem cells (hPSC), ensuring an unlimited supply for β‐cell replacement therapy (reviewed in^[^ ^11^ ^]^). However, these stem cell‐derived β‐cells (SC‐β‐cells) can be functionally immature, with lower glucose‐stimulated insulin‐secretion ability compared to endogenous islets.^[^ ^11^, ^12^ ^]^ Although significant progress has been made in gaining in vitro functional maturity of SC‐β‐cells,^[^ ^13^ ^]^ upon engraftment, a substantial mass of SC‐β‐cells can be lost due to transdifferentiation or dedifferentiation into other cell types, and cell death.^[^ ^12^ ^]^

Porcine pancreatic islets represent another potential source of insulin‐producing cells.^[^ ^14^ ^]^ Given that porcine insulin differs from its human counterpart by only one amino acid (alanine instead of threonine at the carboxy terminus of the B chain) and remains effective in humans, the transplantation of porcine islets in humans (xenotransplantation) emerges as a viable option for β‐cell replacement therapy.^[^ ^14^ ^]^ Porcine islets (PIs) of different developmental stages, including embryonic (EPIs), fetal (FPIs), neonatal (NPIs), and adult (APIs), have been investigated for xenotransplantation.^[^ ^15^ ^]^ Notably, preclinical trials have demonstrated substantially prolonged survival times (up to >603 days) when wild‐type (WT) porcine islets were transplanted into immunosuppressed non‐human primates (NHPs) (**Table** **1**). While these findings are promising, it is essential to acknowledge the considerable variability in the results of these studies, and several of the immunosuppressive regimens employed may not be applicable in a clinical context.

**Table 1:** Use of WT porcine xenoislets in NHPs.

Islets	Recipient NHPs	Immunosuppression regimen	Survival	Reference
NPI	Rhesus monkey	ATG + CVF + rapamycin + anti‐TNF + anti‐CD154 (+Treg)	>603 days	[16]
NPI	Rhesus monkey	CTLA4‐Ig + rapamycin + basiliximab + anti‐CD154	>260 days	[17]
NPI	Rhesus monkey	CTLA4‐Ig + rapamycin + anti‐IL‐2R + anti‐CD40	>203 days	[18]
API	Cynomolgus monkey	Rapamycin + FTY720 + basiliximab + anti‐CD154	>187 days	[19]
NPI	Rhesus monkey	MMF + CTLA4‐Ig + LFA‐3‐Ig + anti‐IL‐2R + anti‐LFA‐1	114 days	[20]
API	Rhesus macaques	anti‐IL‐2R + anti‐CD154 + belatacept + sirolimus + H106	76 days	[21]

API—Adult porcine islets; ATG—Anti‐thymocyte globulin; CD—Cluster of differentiation; CTLA4‐Ig—Cytotoxic T lymphocyte‐associated antigen‐4‐Ig; CVF—Cobra venom factor; Ig—Immunoglobulin; MMF—Mycophenolate mofetil; NPI—Neonatal porcine islets.

The major hurdle in using porcine islets for β‐cell replacement therapy in humans lies in the rejection of xenogeneic islets by the recipient's immune system. Xenogeneic cells or molecules trigger the activation of both the innate and adaptive immune systems, leading to xenograft rejection.^[^ ^22^ ^]^ A crucial factor contributing to this rejection is the inter‐species incompatibility of immune regulatory molecules. In general, xenograft rejection can be classified into 3 categories: i) hyperacute rejection (HAR), occurring within 24 h post‐transplantation; ii) delayed xenograft rejection (DXR), occurring within days to weeks post‐transplantation; and iii) chronic rejection, occurring months to years after xenotransplantation. DXR may stem from acute humoral xenograft rejection (AHXR), cellular xenograft rejection (CXR), and coagulation dysregulation.^[^ ^23^ ^]^ HAR and AHXR predominantly result from the interaction between naturally occurring antibodies in humans and NHPs and porcine xenoantigens.^[^ ^23^ ^]^ CXR involves both innate immune cells (macrophages, NK cells, and neutrophils) and adaptive immune cells (B cells and T cells).

When islets are infused into the portal vein using the Edmonton protocol, they come in direct contact with the recipient's blood. This direct contact may trigger an instant blood‐mediated inflammatory reaction (IBMIR) with kinetics resembling HAR.^[^ ^24^ ^]^ IBMIR is a primary contributor to the loss of islets during the peri‐transplant period.^[^ ^25^ ^]^ In alternative transplantation sites, such as the intraperitoneal space or under the kidney capsule, the islets trigger a localized inflammatory response due to the release of proinflammatory cytokines and “danger” signals upon ischemic damage.^[^ ^26^ ^]^ Both IBMIR and local inflammation entail the recruitment of innate and adaptive immune cells, leading to further damage and subsequent rejection of the xenograft.^[^ ^27^ ^]^

**Figure 1:**
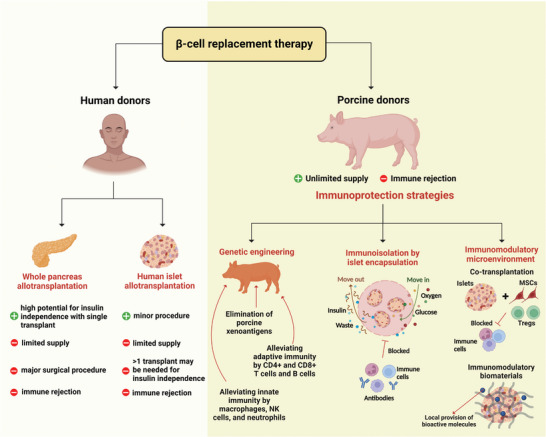
Potential barriers and strategies to counter them in β‐cell replacement therapy. β‐cell replacement therapy offers promising avenues for treating T1DM, with whole human pancreas or pancreatic islet allotransplantation being significant biological treatment options. However, challenges like the limited availability of human pancreata or islets and the need for effective control of allograft rejection with immunosuppressive drugs must be addressed. Porcine islet xenotransplantation has been under consideration as an alternative source for islet transplantation in humans. A major hurdle in islet xenotransplantation is the immune rejection of xenogeneic islets. Different strategies have been devised to circumvent the host immune response against the xenograft and enhance islet survival post‐transplantation, such as genetic modifications in donor pigs to minimize immune recognition and rejection, implementing a physical barrier by encapsulating porcine islets in biocompatible materials, and the creation of an immunomodulatory microenvironment around the islets (created with BioRender.com).

Various strategies are employed to circumvent the immune response of the host against the xenograft. These include genetically modifying donor pigs, immunoisolating islets by diverse encapsulation approaches, and creating an anti‐inflammatory and immunomodulatory microenvironment for (encapsulated) islets (**Figure** **1**). In this review, we explore porcine genetic modifications that have been tested or suggested to increase the survival and functionality of porcine islet xenografts. We have also delved into various immunoisolation and immunomodulation strategies tailored for islets and β‐cells sourced from non‐porcine origins. This exploration is crucial because several challenges in islet allo‐ and xenotransplantation, as well as stem cell‐based β‐cell replacement therapies, are similar. Issues such as foreign body reactions against the encapsulation material or transport properties/limitations are common regardless of the islet source. Therefore, the insights from studies using non‐porcine islet cell sources serve as foundational knowledge for refining immunoprotection strategies for both wild‐type or gene‐modified porcine islets. Moreover, the lessons learned from a wide range of studies using non‐porcine islets can inspire researchers to combine multiple promising therapies, potentially enhancing their efficacy for porcine islets.

## 2 Pharmacological Inhibition of the Instant Blood‐mediated Inflammatory Reaction (IBMIR)

**Figure 2:**
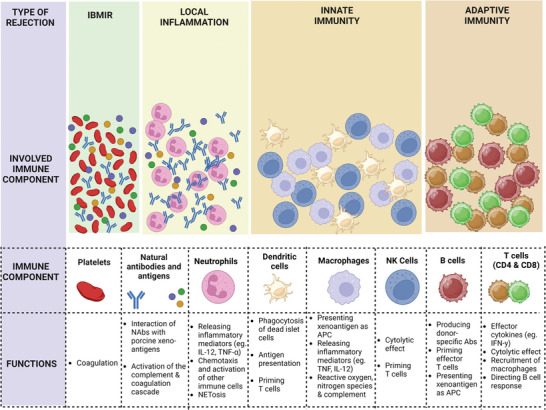
The sequence of host immune responses to islet grafts and the associated immune components. The depicted immune responses include instant blood‐mediated inflammatory reaction (IBMIR), local inflammation, innate immune response, and adaptive immune response (created with BioRender.com).

More than half of the islets infused into the portal circulation are lost during the peri‐transplant period, primarily due to IBMIR.^[^ ^28^ ^]^ IBMIR is an innate immune response observed in autologous, allogeneic, and xenogeneic islet transplantation, specifically targeting the initially free‐floating islets in small liver veins. This response encompasses the activation of complement and coagulation pathways, the stimulation and aggregation of platelets, the release of chemokines and proinflammatory cytokines, and the infiltration of innate immune cells such as dendritic cells, neutrophils, and monocytes (**Figure** **2**).^[^ ^22^, ^28^ ^]^ Both free human or porcine islets exhibit elevated expression of tissue factor (TF), interleukin 8 (IL‐8), and macrophage chemotactic protein 1 (MCP1).^[^ ^24b^ ^]^ TF triggers the coagulation reaction, whereas MCP1 and IL‐8 mediate the recruitment of innate immune cells.^[^ ^24^, ^29^ ^]^ Furthermore, thrombin can enhance the recruitment of immune cells into the transplanted islets.^[^ ^30^ ^]^ Activated immune cells release cytokines and chemokines, amplifying the immune response against the xenograft.^[^ ^30^ ^]^ Interleukin 1‐beta (IL‐1β) and tumor necrosis factor‐alpha (TNF‐α) inflict damage on the islets through nuclear factor kappa B (NF‐κB)‐mediated apoptosis.^[^ ^24b^ ^]^ Additional proinflammatory cytokines and damage‐associated molecular patterns (DAMPs) released by the damaged islets can further escalate local inflammation, promoting the recruitment and activation of immune cells.^[^ ^26^ ^]^ Dendritic cells phagocytize dead or damaged islet cells, presenting alloantigens or xenoantigens on their surface, ultimately priming CD8^+^ and CD4^+^ T cells.^[^ ^27^ ^]^

Numerous approaches have been explored to mitigate the loss of islets during the peri‐transplant period attributed to IBMIR. Interventions such as blocking TF by specific monoclonal antibodies,^[^ ^31^ ^]^ using active site‐inactivated coagulation factor VIIa (FVIIa),^[^ ^31^ ^]^ or selective inhibition of FVIIa by Ro69^[^ ^32^ ^]^ have demonstrated efficacy in vitro, successfully abrogating IBMIR. Similarly, the antioxidant nicotinamide has shown promise in inhibiting TF and MCP1 in isolated human pancreatic islets in vitro.^[^ ^33^ ^]^ Another effective strategy to alleviate IBMIR is targeting inflammatory mediators. For instance, blockade of IL‐1β using anakinra or dual blockade of IL‐1β and TNF‐α using anakinra and etanercept,^[^ ^34^ ^]^ inhibition of NF‐κB by Withaferin A (WA),^[^ ^22^, ^35^ ^]^ and knockout or inhibition of interferon‐gamma (IFN‐γ) inducible protein 10 (IP‐10 alias C‐X‐C motif chemokine ligand 10, CXCL10)^[^ ^36^ ^]^ have proven to significantly alleviate IBMIR and enhance islet survival. Moreover, exogenous administration of activated protein C, an anticoagulant enzyme, showed promise in protecting transplanted islets in diabetic mice.^[^ ^37^ ^]^ Similarly, the administration of cibinetide, an erythropoietin analog,^[^ ^38^ ^]^ or α1‐antitrypsin, a serine protease inhibitor, inhibited IBMIR and improved the engraftment of human islets in mouse models.^[^ ^39^ ^]^ Low molecular weight dextran sulfate (LMW‐DS) has demonstrated the ability to alleviate IBMIR, and a high dose of LMW‐DS not only eliminated leukocyte infiltration but also increased pig islet survival in diabetic mice.^[^ ^29^, ^40^ ^]^ Additionally, a combined administration of high doses of heparin and soluble complement receptor 1 (sCR1), a recombinant complement inhibitor, proved effective in alleviating IBMIR.^[^ ^41^ ^]^ While many of the above‐described strategies have not yet undergone clinical testing, using heparin is the current standard practice to prevent IBMIR in clinical islet transplantation.

IBMIR tends to be more severe in xenogeneic compared to allogeneic and autologous islet transplantation.^[^ ^22^ ^]^ Apart from the presence of TF, IL‐8, and MCP1, porcine islets express — depending on their developmental stage — oligosaccharides like galactose‐α−1,3‐galactose (αGal), N‐glycolylneuraminic acid (Neu5Gc) and Sda, recognized as xenoantigens.^[^ ^14^, ^42^ ^]^ Consequently, porcine islets encounter a robust host immune response in humans and NHPs.^[^ ^28^, ^43^ ^]^ Genetically modifying donor pigs to address molecular incompatibilities has emerged as a potent approach to enhance the survival and efficacy of xenogeneic islet grafts.

## 3 Genetic Modification of Islet Source Pigs

For pig‐to‐NHP or pig‐to‐human islet xenotransplantation, various genetic modifications have been proposed to overcome the challenges posed by IBMIR and subsequent rejection mechanisms.

### 3.1 Eliminating Porcine Xenoantigens

Natural antibodies (NAbs) in humans and non‐human primates (NHPs) pose a significant challenge to islet engraftment and are a primary trigger for IBMIR against porcine islets. The three extensively studied oligosaccharide xenoantigens found on porcine cells but absent in humans, and partially in NHPs, are αGal, Neu5Gc, and Sda.^[^ ^14^, ^42^ ^]^ The enzymes responsible for synthesizing these xenoantigens are α−1,3‐galactosyltransferase (GGTA1) for αGal, cytidine monophosphate‐N‐acetylneuraminic acid hydroxylase (CMAH) for Neu5Gc, and β−1,4‐N‐acetyl‐galactosaminyl transferase 2 (B4GALNT2)/B4GALNT2‐like (B4GALNT2L) for Sda.^[^ ^44^ ^]^ Humans have NAbs against all three major xenoantigens, while NHPs only have NAbs against αGal and Sda.^[^ ^14^, ^45^ ^]^ The presence of NAbs against xenoantigens makes the immune response against porcine xenografts more severe compared to allografts.^[^ ^14^, ^45^ ^]^ Upon the islets encountering host blood, the interaction between xenoantigens and NAbs rapidly activates the complement system.^[^ ^46^ ^]^ Complement activation culminates in the formation of the membrane attack complex (MAC) which is deposited in the cellular lipid bilayer, ultimately causing xenograft rejection through cell lysis and death.^[^ ^46^, ^47^ ^]^

The elimination of porcine xenoantigens by knocking out genes involved in their synthesis has shown promise in improving the engraftment and survival of xenoislets in preclinical trials with NHPs (**Table** **2**). While αGal is strongly expressed on NPIs, its expression is relatively weak on APIs.^[^ ^48^ ^]^ In immunosuppressed diabetic rhesus monkeys, intraportally transplanted *GGTA1*‐KO NPIs exhibited a prolonged survival time of up to 249 days, as opposed to WT NPIs with a maximum survival of 137 days.^[^ ^49^ ^]^ Additionally, the primary nonfunction rate for *GGTA1*‐KO NPIs was reduced to 20%, a significant improvement compared to 80% for WT NPIs.^[^ ^49^ ^]^ However, Martin et al. conducted a study employing a dual‐islet transplant model, wherein both WT and *GGTA1*‐KO NPIs were transplanted into separate liver lobes of the same nondiabetic rhesus monkey. The findings revealed a surprisingly similar host immune response against both types of NPIs at 1 and 2 h post‐transplantation.^[^ ^50^ ^]^ Another study by Samy et al. compared the host immune response against WT and *GGTA1*‐KO NPIs to that against allogeneic islets in the dual‐islet transplant model in the rhesus monkey.^[^ ^51^ ^]^ The host immune response against the xenogeneic WT NPIs was more robust compared to that against allogeneic islets. Despite showing better post‐transplantation engraftment, *GGTA1‐*KO NPIs also exhibited increased antibody deposition and immune cell infiltration compared to allogeneic islets.^[^ ^51^ ^]^ These findings underscore the more intense IBMIR in xenogeneic settings compared to allogeneic scenarios. Furthermore, while the elimination of GGTA1 improved the engraftment of NPIs, achieving the desired graft survival duration may require the elimination of additional xenoantigens.

**Table 2:** Genetic modification strategies for immunoprotection of porcine islets.

Target	Genetic modification		GM pigs	Islet XTx in rodents	Islet XTx in NHPs
**Carbohydrate antigens**					
Deletion of carbohydrate antigens against which humans have natural antibodies	*GGTA1*‐KO	α−1,3‐galactosyltransferase knockout	[115]	–	[49]
	*CMAH*‐KO	cytidine monophosphate‐N‐acetylneuraminic acid hydroxylase knockout	[116]	[117]	–
	*B4GALNT2/2L*‐KO	β−1,4‐N‐acetyl‐galactosaminyl transferase 2 knockout	[30]	–	–
**Complement system**					
Expression of human complement pathway regulatory proteins (CPRPs)	hCD46‐tg	human membrane cofactor protein transgene	[118]	[119]	[61]
	hCD55‐tg	human decay‐accelerating factor transgene	[120]	–	[62, 121]
	hCD59‐tg	human membrane inhibitor of reactive lysis transgene	[122]	–	[62]
	hC1‐INH‐tg	human complement‐regulatory protein C1 inhibitor transgene	[123]	–	–
**Coagulation system**					
Expression of human coagulation‐regulatory genes	hTBM‐tg	human thrombomodulin transgene	[124]	–	–
	hEPCR‐tg	human endothelial protein C receptor transgene	[125]	–	–
	hTFPI‐tg	human tissue factor pathway inhibitor transgene	[126]	–	[63]
	hCD39‐tg	human ectonucleoside triphosphate diphosphohydrolase‐1 transgene	[127]	[128]	[63]
	hCD73‐tg	human ecto‐5′‐nucleotidase transgene	[129]	–	–
**T cells**					
Inhibition of T cell activation	LEA29Y‐tg	affinity‐improved human variant of CTLA4‐Ig transgene	[100a]	[100]	–
	hCTLA4‐Ig‐tg	human cytotoxic T‐lymphocyte associated protein 4 transgene	[130]	–	–
	pCTLA4‐Ig‐tg	porcine CTLA4‐Ig transgene	[131]	–	[63]
	hTRAIL‐tg	human TNF‐related apoptosis‐inducing ligand transgene	[107, 132]	–	–
	PD‐L1‐tg	human programmed cell death 1 ligand 1 transgene	[105]	[106]	–
	*SLA* KO	swine leukocyte antigen knockout	[133]	–	–
	CIITA‐DN‐tg	human dominant‐negative mutant class II transactivator transgene	[97]	–	–
**NK cells and macrophages**					
Inhibition of NK cell and macrophage activation	HLA‐E/β2M‐tg	HLA‐E/human beta 2‐microglobulin transgene	[83]	–	–
	hCD47‐tg	human cluster of differentiation 47 transgene	[76a]	–	–
**Inflammation**					
Expression of anti‐inflammatory proteins	A20‐tg	human tumor necrosis factor alpha‐induced protein 3 (TNFAIP3) transgene	[134]	[68]	–
	hHO‐1‐tg	human heme oxygenase 1 transgene	[135]	[69]	–
	shTNFRI‐Fc‐tg	soluble human TNFRI‐Fc transgene	[135b]	[69]	–

Neu5Gc is present on neonatal, juvenile, and adult porcine islets.^[^ ^52^ ^]^ Since NHPs possess a functional *CMAH* gene,^[^ ^53^ ^]^ studies investigating Neu5Gc elimination are mostly limited to in vitro models. Reports regarding the ability of Neu5Gc to induce an immune response in humans are contradictory. In an in vitro comparison of islets from genetically modified (GM) pigs differing only in *CMAH*‐KO (*GGTA1‐*KO/*CMAH‐*KO/hCD46‐tg versus *GGTA1‐*KO/hCD46‐tg), the absence of Neu5Gc on islet cells did not alter antibody binding.^[^ ^52b^ ^]^ In early clinical trials transplanting WT NPIs into humans, a dominant anti‐αGal response was generated, along with an anti‐Neu5Gc response in some recipients.^[^ ^54^ ^]^ Limited information is available on Sda expression in porcine islets and the impact of Sda elimination on the immunogenicity of xenoislets.^[^ ^55^ ^]^ However, an in vitro study demonstrated a significant reduction in human‐anti‐pig antibody binding with αGal deletion (*GGTA1*‐KO), which was further amplified by Neu5Gc deletion (*CMAH*‐KO) and Sda deletion (*B4GALNT2*‐KO).^[^ ^56^ ^]^ Moreover, NHPs possess some additional NAbs capable of recognizing a currently unidentified xenoantigen on the porcine cells (the fourth antigen) that is exposed upon *CMAH*‐KO.^[^ ^30^ ^]^ Based on the studies involving the currently identified xenoantigens, the knockout of 3 genes responsible for porcine xenoantigens production (3KO) is considered a desirable genetic modification for successful xenotransplantation into humans.^[^ ^57^ ^]^

### 3.2 Inhibition of Complement Activation

The exposure of xenografts to NAbs triggers the activation of the complement system through both classical and alternative pathways.^[^ ^46^, ^58^ ^]^ Complement activation is regulated in part by complement pathway regulatory proteins (CPRPs), which include membrane cofactor protein (MCP/CD46), decay‐accelerating factor (DAF/CD55), and membrane inhibitor of reactive lysis (CD59).^[^ ^59^ ^]^ Transgenic expression of human CPRPs in GM donor pigs has been shown to inhibit the recipient's complement activation and support xenograft survival after pig‐to‐human xenotransplantation.^[^ ^60^ ^]^ To assess the expediency of transgenic expression of hCD46 (hCD46‐tg) in intraportal islet xenotransplantation, APIs from WT, hCD46‐tg, or *GGTA1*‐KO pigs were transplanted into immunosuppressed diabetic monkeys.^[^ ^61^ ^]^ Although hCD46 expression did not reduce the degree of IBMIR, hCD46‐tg APIs exhibited a longer survival time and an extended duration of insulin independence (87–396 days) compared to the 5–36 days observed in the case of WT APIs. In contrast, *GGTA1‐*KO APIs exhibited similar performance to WT APIs, consistent with the fact that APIs have low levels of αGal.^[^ ^61^ ^]^

Hawthorne et al. reported that GM NPIs (*GGTA1‐*KO/hCD55‐tg/hCD59‐tg/human 1,2‐fucosyltransferase (hHT)‐tg) displayed minimal signs of IBMIR and an absence of thrombosis compared to WT NPIs, which were rapidly lost after transplantation due to extensive IBMIR.^[^ ^62^ ^]^ A similar conclusion was drawn in another study comparing *GGTA1‐*KO/hCD46‐tg APIs with WT or hCD46‐tg APIs.^[^ ^63^ ^]^ Moreover, incubation of *GGTA1‐*KO/hCD46‐tg NPIs with whole human blood in vitro exhibited reduced complement activation but accelerated coagulation compared to WT NPIs.^[^ ^64^ ^]^ Recently, two distinct preclinical trials were conducted in a dual‐islet transplantation model in rhesus monkeys to compare *GGTA1*‐KO/hCD46‐tg with *GGTA1‐*KO NPIs.^[^ ^65^ ^]^ Initial post‐transplantation assessments at 1 h showed no significant differences in platelet and antibody deposition, complement activation, and neutrophil infiltration. However, at 24 h post‐transplantation, *GGTA1*‐KO/hCD46‐tg NPIs exhibited significantly lower platelet deposition and neutrophil infiltration, with no difference in antibody deposition and complement activation.^[^ ^65a^ ^]^ The observed antibody deposition and complement activation in these studies suggest the potential presence of other xenoantigens, reinforcing the notion of knocking out additional xenoantigens and expressing human CPRPs to counteract IBMIR. Moreover, the presence of platelet aggregation and coagulation activation emphasizes the necessity of intervening in the coagulation pathway as well.

### 3.3 Prevention of Coagulation Dysregulation

Under normal physiological conditions, thrombomodulin (TBM) binds thrombin, altering its substrate specificity from coagulation factors like fibrinogen to protein C, thereby inducing an anticoagulation effect.^[^ ^66^ ^]^ While not explicitly reported, it is likely that porcine islets do not express TBM.^[^ ^14^ ^]^ Furthermore, the porcine TBM‐human thrombin complex cannot efficiently activate human protein C.^[^ ^66^ ^]^ Therefore, the transgenic expression of human TBM (hTBM‐tg) in porcine islets could prove beneficial in inhibiting the coagulation pathway. Other genes of interest for modulating the human coagulation pathway include endothelial protein C receptor (EPCR), tissue factor pathway inhibitor (TFPI), and the thromboregulatory enzyme CD39.^[^ ^14^ ^]^ Incubating islets from hCD39‐tg mice with human blood exhibited a delayed initiation of coagulation reaction compared to WT mouse islets.^[^ ^67^ ^]^ Conversely, when *GGTA1*‐KO/hCD46‐tg/hCD39‐tg NPIs were incubated with human blood, no difference in coagulation time was observed compared to *GGTA1*‐KO/hCD46‐tg NPIs.^[^ ^64^ ^]^ Transplanting GM APIs (*GGTA1‐*KO/hCD46‐tg/hTFPI‐tg/CTLA4‐Ig or *GGTA1*‐KO/hCD46‐tg/hCD39‐tg/hTFPI‐tg/CTLA4‐Ig) into immunosuppressed diabetic cynomolgus monkeys demonstrated significantly lower IBMIR at 2 h post‐transplantation compared to WT or hCD46‐tg APIs.^[^ ^63^ ^]^ However, the long‐term survival of APIs exhibited high variability,^[^ ^63^ ^]^ advocating for further studies and preclinical trials to identify an optimal combination of genetic modifications.

### 3.4 Antiinflammatory Strategies

The expression of transgenes aimed at reducing inflammatory responses has also been proposed. For example, the ubiquitin‐editing enzyme A20 is a natural suppressor of islet inflammation. NPIs expressing hA20‐tg demonstrated a reduction in the inflammatory response against islets by inhibiting the activation of NF‐κB by TNF‐α in vitro.^[^ ^68^ ^]^ When these hA20‐tg NPIs were transplanted into immunodeficient mice, they exhibited improved function and extended survival.^[^ ^68^ ^]^ Additionally, anti‐inflammatory molecules such as soluble TNF‐α receptor (sTNFR) and heme oxygenase‐1 (HO‐1) have shown promise. APIs derived from sTNFR‐tg or HO‐1‐tg pigs demonstrated a decreased infiltration of host immune cells and an increased survival time post‐transplantation in mice.^[^ ^69^ ^]^

### 3.5 Modulating the Innate Cellular Immune Response

#### 3.5.1 Macrophages

**Figure 3:**
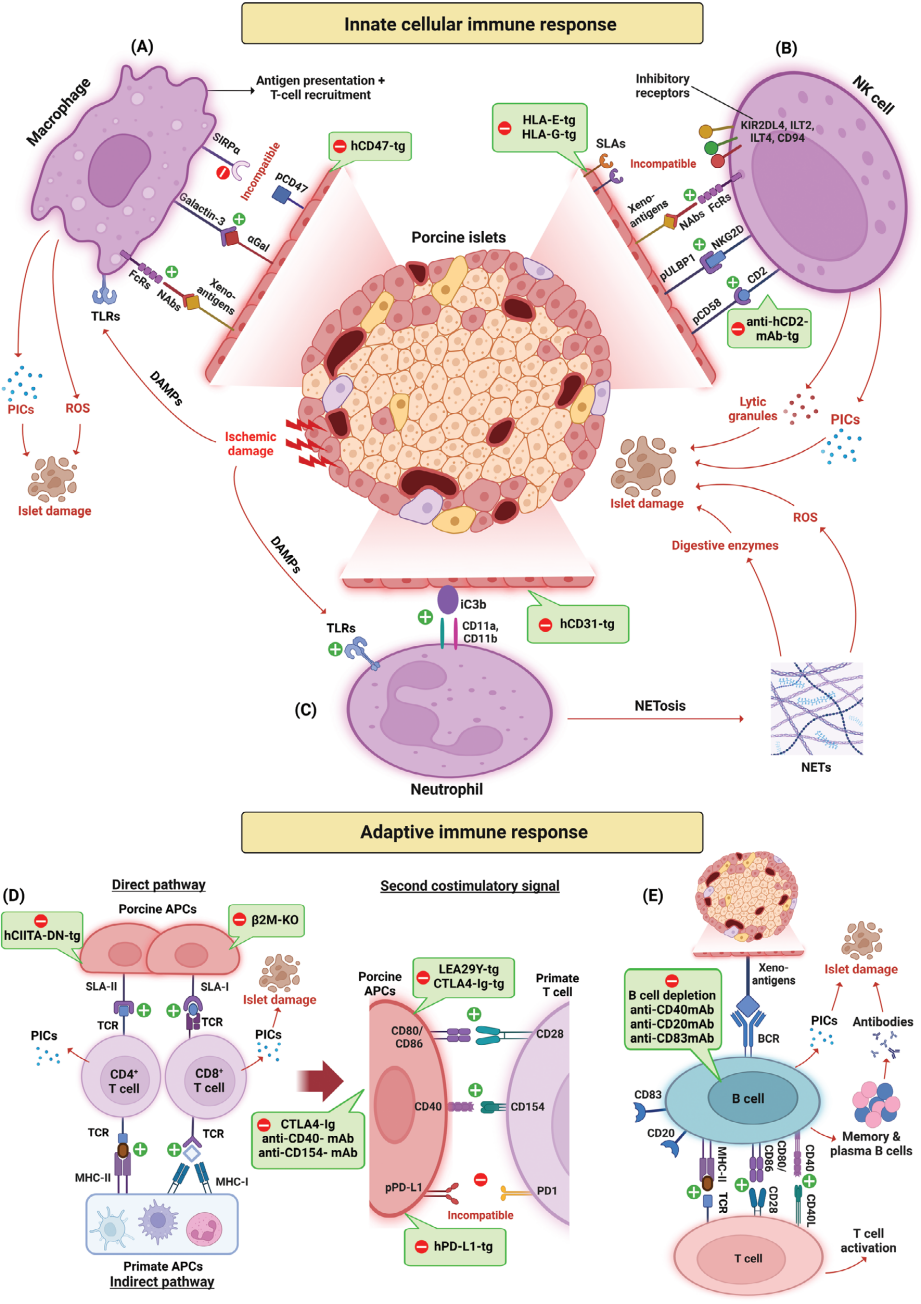
The innate cellular and adaptive immune response in islet xenotransplantation. Pathways involved in and suggested solutions to counter the activation of A) macrophages, B) NK cells, C) neutrophils, D) T cells, and E) B cells. Abbreviations: NAbs: natural antibodies; FcRs: Fc receptors; TLRs: Toll‐like receptors; PICs: proinflammatory cytokines; ROS: reactive oxygen species; SLAs: swine leukocyte antigens; NK cell: natural killer cell; DAMPs: damage‐associated molecular patterns; BCRs: B cell receptor; mAb: monoclonal antibodies (created with BioRender.com).

The innate cellular immune response against the xenograft is predominantly orchestrated by macrophages, natural killer (NK) cells, and neutrophils (**Figure** **3**). In a rodent model where porcine islets were transplanted, macrophages were observed as one of the first immune cell types to infiltrate the xenograft.^[^ ^28b^ ^]^ They play a crucial role not only in CXR but also in NAb‐mediated HAR and DXR.^[^ ^46^, ^70^ ^]^ Following xenogeneic islet cell transplantation, macrophages can be activated through interaction between i) antigen‐antibody immune complexes and Fc receptors (FcRs) on macrophages^[^ ^71^ ^]^; ii) galectin‐3 (abundant on macrophages) and αGal on porcine cells^[^ ^71a^ ^]^; iii) macrophages and other infiltrating immune cells such as neutrophils, NK cells, and Th1 cells^[^ ^72^ ^]^; and (iv) DAMPs released by damaged islets and Toll‐like receptors (TLRs).^[^ ^73^ ^]^ Macrophages contribute to xenograft destruction either through direct cytotoxic effects or by facilitating T‐cell recruitment.^[^ ^70^, ^71^, ^74^ ^]^

Macrophages distinguish between self and non‐self cells through an inhibitory signaling pathway involving CD47 and signal regulatory protein alpha (SIRPα).^[^ ^75^ ^]^ The interaction of CD47 on target cells with SIRPα on macrophages and neutrophils generates a “do not eat me” signal, identifying cells expressing CD47 as “self” and providing them protection.^[^ ^75a^ ^]^ However, porcine CD47 is not compatible with human or baboon SIRPα, leaving porcine cells unprotected when encountering human macrophages in vivo^[^ ^76^ ^]^ or in vitro.^[^ ^77^ ^]^ The transgenic expression of human CD47 (hCD47) in pigs has proven to be protective against macrophages, with hCD47‐trangenic (hCD47‐tg) porcine hematopoietic cells or skin xenografts outperforming control xenografts in baboon models.^[^ ^76^ ^]^ Similarly, following intraportal transplantation in mice, rat islets expressing mouse CD47 were protected from disruption caused by IBMIR, displaying enhanced engraftment and function compared to control islets.^[^ ^78^ ^]^ In a recent study, Ghimire et al. found that CD47 inhibits insulin release from β‐cells by deactivating the cell division control protein 42 homolog (Cdc42) while blocking CD47 enhances glucose‐stimulated insulin secretion (GSIS) in mouse and human islets.^[^ ^79^ ^]^ Furthermore, syngeneic transplantation of *Cd47* ^−/−^ mouse islets exhibited superior efficacy in normalizing blood glucose values compared to *Cd47* ^+/+^ mouse islets in a preclinical islet transplant model. Treating non‐obese diabetic (NOD) mice with a CD47‐blocking monoclonal antibody (Miap301) improved glycemic control and delayed the onset of overt diabetes.^[^ ^79^ ^]^ Nevertheless, the potential impact of hCD47 expression in porcine islets on pig‐to‐NHP islet xenotransplantation and the subsequent survival and function of transplanted islets remains an unexplored area.

#### 3.5.2 Natural Killer Cells

Natural killer (NK) cells play a pivotal role in islet destruction and are activated by the abundant proinflammatory cytokines released during IBMIR and by the macrophages.^[^ ^80^ ^]^ The activated NK cells contribute to xenograft destruction either through their direct interaction with xenogeneic cells or via antibody‐dependent cellular cytotoxicity (ADCC).^[^ ^71a^ ^]^ In direct interaction, NK cells engage with their target cells through activating or inhibitory receptors. The activating NK cell receptors NKG2D and CD2 interact with pULBP1 and a CD58 ortholog on porcine cells, respectively, triggering NK cell activation. Activated NK cells release lytic particles and cytokines (TNF‐α and IFN‐γ), leading to the lysis of the target cells.^[^ ^81^ ^]^ On the other hand, inhibitory NK cell receptors (KIR2DL4, ILT2, ILT4, and CD94/NKG2A) on human NK cells typically bind major histocompatibility complex (MHC) class I molecules, such as human leukocyte antigen E (HLA‐E) and HLA‐G, generating an inhibitory signal. However, these receptors cannot effectively interact with swine leukocyte antigens (SLAs) and, as a result, an inhibitory signal is not generated by porcine cells, ultimately leading to cell lysis.^[^ ^82^ ^]^ In ADCC, Fc receptors (FcRs) on NK cells interact with antibodies deposited on xenogeneic cells, triggering the release of lytic granules and apoptosis of the target cells.^[^ ^82^ ^]^

One potential strategy to mitigate the impact of human NK cells on porcine cells involves the transgenic expression of NK cell inhibitory ligands, such as HLA‐E and HLA‐G, in donor pigs. In vitro studies have shown that porcine endothelial cells expressing HLA‐E are protected against the action of human NK cells.^[^ ^83^ ^]^ Rao et al. took a step further by generating *GGTA1‐*KO/HLA‐G1‐tg pigs. Their findings indicated that *GGTA1‐*KO/HLA‐G1‐tg porcine fibroblasts were protected against the action of T cells, macrophages, and NK cells when compared to WT porcine fibroblasts.^[^ ^84^ ^]^ Moreover, G*GTA1*‐KO/HLA‐G1‐tg APIs transplanted under the kidney capsule of diabetic nude mice restored normoglycemia and exhibited a longer survival time than their WT counterparts.^[^ ^84^ ^]^ However, this study was terminated at day 32, and the long‐term survival of APIs, as well as the in vivo host immune response, were neither accessed nor reported.

#### 3.5.3 Neutrophils

Neutrophils are recruited into the xenograft in response to IBMIR, proinflammatory cytokines released by macrophages, and DAMPs. They can also also independently interact with xenogeneic cells.^[^ ^59a^ ^]^ Additionally, neutrophils can be activated by the interaction of their CD11a and CD11b receptors with iC3b protein, a component of the complement system, deposited on porcine cells.^[^ ^59a^ ^]^ Upon exposure to various stimuli, activated neutrophils undergo “NETosis”, a form of programmed cell death.^[^ ^85^ ^]^ NETosis results in the formation of neutrophil extracellular traps (NETs) containing serine proteases and antibacterial peptides. NETs can generate reactive oxygen species (ROS) and digestive enzymes that damage xenograft cells.^[^ ^85^, ^86^ ^]^ CD31, also known as platelet EC adhesion molecule 1 (PECAM1), expressed on hemopoietic and endothelial cells, functions as an inhibitor of mitochondrial apoptosis.^[^ ^87^ ^]^ Interestingly, porcine cells expressing hCD31 suppress NETosis and its resultant cytotoxicity.^[^ ^88^ ^]^ Therefore, the expression of hCD31 in pigs can be utilized to inhibit neutrophil‐mediated xenograft rejection.

### 3.6 Alleviating the Adaptive Immune Response

#### 3.6.1 T Cells

The islets that manage to evade IBMIR and HAR may still face acute cellular rejection by T cells.^[^ ^28b^ ^]^ T cells play a pivotal role in xenograft rejection, with both CD4^+^ and CD8^+^ cells capable of infiltrating the xenograft.^[^ ^89^ ^]^ There are 2 pathways of T cell activation: the direct pathway and the indirect pathway (Figure 3).^[^ ^28b^ ^]^ In the direct pathway, intact SLA‐I and SLA‐II on porcine APCs (such as endothelial cells and resident dendritic cells) interact directly with the host's T cell receptors (TCRs) on CD8^+^ and CD4^+^ T cells, respectively.^[^ ^90^ ^]^ In the indirect pathway, peptides derived from porcine tissue are presented by host APCs to host CD8^+^ and CD4^+^ T cells.^[^ ^90^ ^]^ TCR‐induced T cell activation is amplified by costimulatory signals primarily generated by the interaction between CD154 or CD28 on the host's T cells and CD40 or CD80/CD86 on xenogeneic APCs, respectively.^[^ ^91^ ^]^ These costimulatory signals are critical for full T cell activation, proliferation, and differentiation.^[^ ^92^ ^]^ In contrast, the interaction between programmed cell death protein 1 (PD‐1) on CD4^+^ and CD8^+^ T cells and human programmed death ligand‐1 (hPD‐L1) on target cells generates a coinhibitory signal, blocking T cell proliferation and activation.^[^ ^93^ ^]^ However, hPD‐1 or hPD‐L1 are not compatible with their porcine counterparts.^[^ ^94^ ^]^

T cells not only exert their cytotoxic effect by producing inflammatory cytokines but also amplify the cellular xenograft rejection by recruiting and activating macrophages and directing B cell response.^[^ ^28b^ ^]^ Based on the above‐described mechanisms, the T cell‐mediated cytotoxicity against the xenograft can be alleviated by:

##### Removing of SLAs

This can suppress T‐cell responses against porcine xenografts. However, complete removal of SLAs may have adverse effects, as SLAs also have protective immune functions in pigs.^[^ ^95^ ^]^ Therefore, instead of complete removal, reducing SLA expression can mitigate T cell‐mediated cytotoxicity and avoid potential adverse effects associated with complete SLA inhibition.^[^ ^96^ ^]^ Transgenic pigs expressing a human dominant‐negative mutant class II transactivator transgene (hCIITA‐DN‐tg) exhibit significantly reduced SLA‐II expression on APCs.^[^ ^97^ ^]^ Conversely, the knockout of porcine beta 2‐microglobulin (β2M) abrogates SLA‐I expression in porcine cells.^[^ ^95^ ^]^

##### Suppression of Costimulatory Signals

Blocking costimulatory pathways can be achieved by using specific monoclonal antibodies or by generating GM pigs expressing immunomodulatory molecules. For instance, the CD40–CD154 costimulatory signal can be blocked by anti‐CD154 or anti‐CD40 monoclonal antibodies.^[^ ^98^ ^]^ The CD28‐CD80/CD86 costimulatory signal can be inhibited by soluble human cytotoxic T‐lymphocyte antigen 4‐immunoglobulin (CTLA4‐Ig) or by generating GM pigs expressing CTLA4‐Ig.^[^ ^99^ ^]^ GM pigs expressing islet‐specific LEA29Y, a high‐affinity variant of CTLA4‐Ig, have been generated to suppress the costimulatory signals.^[^ ^100^ ^]^ When transplanted into diabetic mice with a humanized immune system, LEA29Y‐tg NPIs not only reversed hyperglycemia in 70% of the recipients but also exhibited a prolonged survival period exceeding 6 months.^[^ ^100b^ ^]^ In contrast, WT NPIs faced rejection even before reaching maturity.^[^ ^100b^ ^]^ CD2 is an activating receptor on T cells required for their interaction with APCs.^[^ ^101^ ^]^ Nottle et al. produced pigs with *GGTA1* knockout and transgenic expression of anti‐hCD2‐mAb.^[^ ^102^ ^]^ The islets from these pigs may be more likely to be tolerated by the human/NHP immune system.

##### Transgenic Expression of Inhibitory Signaling Molecules

Xenografts from GM pigs expressing hPD‐L1 may evade human T‐cell‐mediated immune rejection.^[^ ^103^ ^]^ Notably, human islet‐like organoids expressing PD‐L1 have demonstrated prolonged survival, exceeding 50 days, in immunocompetent diabetic mice.^[^ ^104^ ^]^ Similarly, cells isolated from hPD‐L1‐tg pigs exhibited reduced proliferation and cytotoxicity of human CD4^+^ T cells.^[^ ^105^ ^]^ In a recent study by Lei et al., NPIs from WT or hPD‐L1‐tg pigs were transplanted into humanized diabetic mice.^[^ ^106^ ^]^ After 16 weeks, recipients transplanted with hPD‐L1‐tg NPIs showed a significantly higher rate of normoglycemia (50% vs 0%), elevated plasma C‐peptide levels, and decreased infiltration of immune cells within the graft compared to the WT group.^[^ ^106^ ^]^ Another potential factor in suppressing T cell‐mediated immune rejection is the tumor necrosis factor‐related apoptosis‐inducing ligand (TRAIL). However, it's important to note that only dendritic cells from pigs expressing human TRAIL demonstrated an antiproliferative effect on T cells.^[^ ^107^ ^]^ This raises questions about the broader application of this genetic modification in porcine islet xenotransplantation.

#### 3.6.2 B Cells

Interaction between B cell receptor (BCR) and xenoantigens on porcine cells initiates B cell activation, transforming them into potent APCs (Figure 3).^[^ ^108^ ^]^ In their role as APCs, B cells present MHC‐II‐peptide complexes that interact with TCR on T cells. Additionally, the interaction of CD40 and CD80/86 on B cells with CD40L and CD28 on T cells, respectively, triggers the differentiation of B cells into antibody‐producing plasma cells or memory B cells and T cells into effector T cells.^[^ ^109^ ^]^ The potential outcomes of B cell activity include graft rejection through the production of xenoantigen‐specific antibodies, priming of effector T cells, antigen presentation, and cytokine production.^[^ ^110^ ^]^ In immune‐competent diabetic C57BL/6 (B6) mice transplanted with encapsulated NPIs, a transient decrease in blood glucose level was observed at 1‐week post‐transplantation, but hyperglycemia returned to pre‐transplantation levels by 2 weeks, indicating rejection of the NPIs. Notably, an overgrowth of CD4^+^ T cells, macrophages, and B cells occurred on the encapsulated islets, with a significant correlation between cellular overgrowth and islet cell death.^[^ ^111^ ^]^ Conversely, *Rag1*‐knockout diabetic B6 mice lacking mature B and T lymphocytes transplanted with encapsulated NPIs exhibited normoglycemia for up to 100 days post‐transplantation without any immune cell growth.^[^ ^111^ ^]^ Hence, strategies such as B cell depletion or modulation of B cell function using antibodies like anti‐CD40, anti‐CD20, or anti‐CD83 can prolong xenograft survival.^[^ ^98^, ^112^ ^]^ In rat‐to‐mouse islet xenotransplantation, combined B and T cell depletion inhibited donor‐specific antibody production, leading to indefinite xenograft survival.^[^ ^113^ ^]^ However, in pig‐to‐mouse islet xenotransplantation, the same approach did not achieve long‐term survival islet survival, and islets were lost due to a combined response of B and T cells.^[^ ^110^, ^114^ ^]^

## 4 Biomaterial‐Assisted Encapsulation of Islets

**Figure 4:**
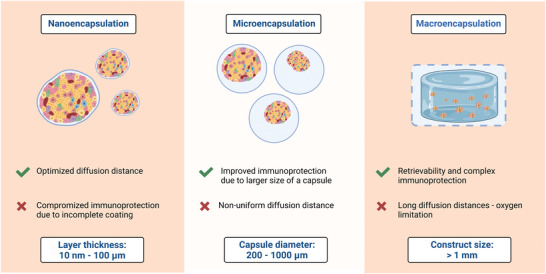
A comparison of nano‐, micro‐, and macroencapsulation strategies (created with BioRender.com).

Immunoisolation typically involves encapsulating one or more islets within a selectively permeable biocompatible material or a device featuring a permselective element. This design permits the free exchange of small molecules like nutrients, oxygen, and hormones between the encapsulated cells and their surroundings while preventing the passage of immune cells and large immunoglobulins.^[^ ^136^ ^]^ Thus, the permselective material or element acts as a physical barrier, protecting the encapsulated islets from the host´s immune system, and vice versa, ideally obviating the need for systemic immunosuppression. Isolation of islets, however, deprives them of their native environment, including vascularization, innervation, and extracellular matrix (ECM). Therefore, in addition to immunoisolation, crafting a biomimetic microenvironment for the islets and facilitating efficient vascularization should be considered while formulating islet encapsulation strategies.^[^ ^137^ ^]^ Various encapsulating materials, sometimes used in combinations, have been employed to create an artificial microenvironment for isolated islets. Hydrogels, comprising a densely cross‐linked hydrophilic polymer network with elevated water content, stand out as the most extensively utilized materials^[^ ^136^ ^]^ Hydrogel networks typically possess mesh sizes within the range of several tens of nanometers, making them ideal as perm‐selective elements. Additionally, alternative materials, primarily polymers, have been utilized to modulate permeability and enhance the mechanical stability of the encapsulation layer, matrix, or device. The encapsulation of pancreatic islets can be categorized into nanoencapsulation, microencapsulation, and macroencapsulation^[^ ^138^ ^]^ (**Figure** **4**). In the subsequent discussion, we will elucidate the fundamental principles of these three approaches, accompanied by illustrative examples.

Microcapsules have emerged as the most studied islet encapsulation platform to date. Furthermore, the majority of xenotransplantation studies involving porcine islets in NHPs have predominantly employed the microencapsulation approach. Thus, our focus will initially center on delineating microencapsulation strategies, along with their inherent advantages and disadvantages. Subsequently, we will examine how the development of nano‐ and macroencapsulation techniques has aimed to overcome certain disadvantages of microencapsulation and evaluate the efficacy of these alternative methods. Our exploration involves a wide array of studies involving native allogeneic, syngeneic, and xenogeneic islets, as well as stem cell‐derived islet cells and spheroids or pseudo‐islets/organoids derived from any of the aforementioned sources. This comprehensive review provides insights into their potential suitability for encapsulating porcine islets and facilitating their subsequent xenotransplantation.

### 4.1 Microencapsulation

In microencapsulation, a single islet or a small cluster of islets is enclosed within a spherical microcapsule made of a biocompatible perm‐selective material.^[^ ^139^ ^]^ Various materials have been utilized, such as cellulose, agarose, collagen, chitosan, poly(ethylene glycol) (PEG), gelatin, and poly(2‐hydroxyethyl methacrylate). Alginate, in particular, has gained popularity as a polymer of choice for microencapsulation and has undergone clinical trials as well.^[^ ^136^, ^140^ ^]^ In most studies, alginate capsules are additionally coated with a thin polycation layer, such as poly‐L‐ornithine (PLO), poly‐L‐lysine (PLL), polyallylamine, or polyvinylamine. This additional coating enhances the permselectivity of the capsule and ensures its mechanical stability. Subsequently, this polycation layer is then covered by an extra outer layer of alginate (reviewed in^[^ ^140^ ^]^). Numerous methods have been developed and tested for generating microcapsules, with droplet generators being the most prevalent.^[^ ^141^ ^]^ However, the variability in droplet size has been a significant challenge. To address this issue, optimized air droplet generators and electrostatic droplet generators have been devised, providing enhanced uniformity in capsule size.^[^ ^139^, ^141^, ^142^ ^]^

Reversing hyperglycemia with microencapsulated islets demands a substantially large quantity that exceeds the manageable size for administration into the liver via the portal vein.^[^ ^143^ ^]^ This notable excess in the volume of encapsulation material compared to actual islet mass results from the rather large size of the capsules and the overproduction of empty capsules by encapsulation devices. It also increases the risk of thrombosis and vascular clogging following transplantation into the clinically proven site for free islet transplantation. To address this significant drawback in the clinical use of microencapsulated islets, advanced purification systems have been developed that can effectively separate empty microcapsules from the mixture using magnetic separation techniques.^[^ ^144^ ^]^ Furthermore, the challenge of controlling the localization and retrieval of microencapsulated islets after transplantation persists.^[^ ^140^ ^]^ This aspect requires attention and innovative solutions to optimize the efficacy and precision of microencapsulated islet transplantation procedures.

The first successful clinical trial of microencapsulated allogeneic islets was reported in 1994 by Soon‐Shiong et al. who used purified alginate with a high guluronic acid content to coat the islets.^[^ ^145^ ^]^ The islets were transplanted into the peritoneum of an immunosuppressed patient with diabetes, resulting in insulin independence for a remarkable 9‐month period. In a subsequent study, Calafiore et al. transplanted alginate‐PLO‐alginate microencapsulated allogeneic islets into the peritoneal cavity of individuals with diabetes, achieving a reduction in exogenous insulin requirements for several weeks post‐transplantation without the use of immunosuppression.^[^ ^146^ ^]^ Similarly, Tuch et al. encapsulated allogeneic islets in barium‐crosslinked alginate microcapsules and transplanted them into the peritoneal space of four diabetes patients without employing immunosuppression.^[^ ^147^ ^]^ Although the patients experienced a reduction in blood glucose levels, no significant change was observed in exogenous insulin requirements.^[^ ^147^ ^]^

Elliott et al. demonstrated the safety of repeated intraperitoneal xenotransplantation of NPIs encapsulated in alginate‐polyornithine‐alginate (APA) in diabetic primates.^[^ ^148^ ^]^ The study showed a significant reduction in insulin dose requirements in the majority of diabetic cynomolgus monkeys subjected to this procedure.^[^ ^148^ ^]^ In a clinical trial involving xenogeneic porcine islet transplantation into human patients with diabetes, APA microencapsulated NPIs (DIABECELL^®^, Living Cell Technologies (LCT)) were transplanted into eight patients. Six of these patients experienced a reduced exogenous insulin requirement for up to 8 months, however, complete and long‐term insulin independence was not achieved.^[^ ^140^, ^149^ ^]^ A notable aspect of this trial was the demonstration of the microbiological and virological safety of NPIs, as evidenced by the absence of pathogen transmission from porcine islets to human recipients.^[^ ^150^ ^]^ As of now, none of the clinical trials using free or encapsulated porcine islets have achieved long‐term insulin independence in patients with T1DM (examples are provided in **Table** **3**).

**Table 3:** Human clinical trials using free and microencapsulated porcine islets.

Islets (pig breed)	Encapsulating material	Site	Immuno‐suppression	Prominent findings	Reference
Free NPIs (Xeno‐I)	–	Intraportal	Yes	Reduced insulin requirement, no long‐term control	[151]
Free FPIs (Swedish Landrace)	–	Intraportal or kidney capsule	Yes	No improvement in glycemic control, porcine C‐peptide (PcP) ≤ 0.4 nmol	[54a]
Encapsulated NPIs (Auckland Island)	APA	Peritoneum	None	Reduced unaware hypoglycemic events, minimal changes in insulin requirement, transplant estimated function (TEF) ≥ 0.5 (full graft function)	[152]
Encapsulated NPIs (Auckland Island)	APA	Peritoneum	None	Reduced unaware hypoglycemic events, minimal changes in insulin requirement, TEF ≥ 0.5 (full graft function)	[150a]
Encapsulated NPIs (Cross‐White)	L‐lysin‐alginate	Peritoneum	None	Over 9 years of survival, low‐level insulin production, PcP ≤ 9.5 ng mL^−1^ (4 months); 0.6 ng mL^−1^ (11 months)	[153]

While microencapsulation of islets prevents their direct interaction with the immune system, the host's inflammatory response to foreign bodies can still be triggered, leading to fibrosis around the transplanted capsules. This fibrosis may disrupt nutrient supply, resulting in the subsequent necrosis of the encapsulated islets.^[^ ^154^ ^]^ To address this issue, co‐encapsulating anti‐inflammatory drugs such as curcumin, pentoxifylline (PTX), or dexamethasone with islets has been shown not only to enhance efficiency but also to inhibit inflammatory response around the microcapsule in vivo.^[^ ^155^ ^]^ In a recent study by Kim et al., alginate‐microencapsulated porcine islets were coated with dexamethasone‐21‐phosphate (dexa) dissolved in 1% chitosan and transplanted into diabetic mice.^[^ ^156^ ^]^ The dexa‐chitosan coating did not impact the viability and functionality of porcine islets and maintained normoglycemia for an extended duration of 231 days. Moreover, the peri‐capsular inflammatory response was significantly reduced around the dexa‐chitosan‐coated microcapsules compared to the non‐coated ones.^[^ ^156^ ^]^ Additionally, it has been noted that the purity of alginate is linked to its degree of biocompatibility, whereby less purified alginate tends to induce more fibrosis around the capsules.^[^ ^157^ ^]^ Beyond the material, other capsule properties, such as their size have been reported to influence the fibrotic response.^[^ ^158^ ^]^ For instance, when encapsulated rat islets were transplanted into diabetic mice, larger capsules with diameters greater than 1.5 mm induced less fibrosis compared to smaller microcapsules with diameters of 0.5 mm, regardless of the biomaterials used.^[^ ^159^ ^]^ Further discussion on immunomodulating microcoatings is provided in Section 5 of the manuscript.

### 4.2 Nanoencapsulation

Several limitations associated with microencapsulation approaches can be addressed through the engineering of ultrathin polymer films directly onto the islet surface. These nano‐ or conformal coatings, with a film thickness ranging from several nanometers to several tens of micrometers, adapt to the size of the islets, resulting in coatings of uniform thickness independent of the islet diameter. One notable advantage of these ultrathin immune barriers, conforming to the islet surface, is the reduction of the distance that passive diffusion must traverse, ensuring efficient transport of nutrients and hormones into and out of the islets.^[^ ^160^ ^]^ Moreover, due to the reduced total transplant volume, nanoencapsulated islets are compatible with intraportal transplantation^[^ ^161^ ^]^ and do not cause blood vessel clogging after transplantation into this clinically proven site.^[^ ^162^ ^]^ Coatings can be further functionalized to protect the encapsulated islets from IBMIR.^[^ ^163^ ^]^ However, the ultra‐small size and relatively free movement of the capsules pose challenges for their desired removal from the host system,^[^ ^140^, ^164^ ^]^ similar to microencapsulated islets. Nanoencapsulation of islets can be broadly categorized into two major classes: i) decoration with thin PEG‐based coatings (PEGylation) and ii) layer‐by‐layer (LBL) assembly, as reviewed in.^[^ ^158^ ^]^

#### 4.2.1 PEG‐Based Coatings

PEG and its derivatives are the frequently used biomaterials for the conformal coating of islets.^[^ ^164^ ^]^ Such coatings can be achieved through interfacial photopolymerization, such as under ultraviolet/visible light in the presence of a photo‐initiator on the islet surface. The thickness of the coating can be controlled by adjusting the PEG composition and the conditions of polymerization.^[^ ^158^ ^]^ Hill et al. tested this approach for xenotransplantation of PEG‐coated APIs into diabetic rats, achieving restoration of normoglycemia.^[^ ^165^ ^]^ However, long‐term euglycemia or insulin independence was not sustained, and the animals returned to hyperglycemia after 60–70 days.^[^ ^165^ ^]^ In a pilot study, Scharp et al. subcutaneously transplanted PEG‐coated allogeneic islets into diabetic baboons, with 3 out of 5 recipients maintaining insulin independence for 14–20 months.^[^ ^166^ ^]^ In a subsequent study limited to 3–6 months duration, most diabetic baboon recipients showed a significant reduction in blood glucose levels and daily insulin requirement, with 7 out of 16 recipients remaining insulin‐independent during the trial period.^[^ ^166^ ^]^ Novocell, later Viacyte, conducted a human clinical trial that involved the subcutaneous transplantation of conformally PEG‐coated allogeneic islets into 12 patients with T1DM.^[^ ^167^ ^]^ However, the trial was terminated shortly after initial observations indicated a limited efficacy of this approach in the first 2 recipients.^[^ ^164^, ^168^ ^]^

Tomei et al. presented an improved approach for conformal coating. They developed a new microfluid method to generate thin (a few tens of micrometers) and continuous PEG coatings that fully covered the islets.^[^ ^169^ ^]^ This approach significantly prolonged the survival of encapsulated islets compared to naked islets in an allogeneic mouse model in the absence of immunosuppression.^[^ ^169b^ ^]^ To achieve even thinner nano‐ or single‐layer coatings, PEG terminated with a protein‐reactive functional group can be chemically grafted to the islet surface using membrane surface proteins of peripheral islet cells as conjugation sites, as demonstrated for porcine islets by Contreras et al.^[^ ^170^ ^]^ In another study, the covalent grafting of PEG molecules onto the collagen capsule surrounding the islets was found to effectively prevent the activation of immune cells but was unable to fully prevent the infiltration of cytotoxic molecules into the islets.^[^ ^171^ ^]^ Alternatively, hydrophobic interactions can be employed to create nano‐thin PEG coatings on the islet surface.^[^ ^158^ ^]^ For instance, lipid‐conjugated PEG can rapidly conjugate with the islet cells' phospholipid bilayer membrane through hydrophobic interaction, forming a coating.^[^ ^172^ ^]^ Intraportal infusion of xenogeneic islets coated with lipid‐conjugated PEG resulted in short‐term graft success.^[^ ^173^ ^]^

Despite their advantages, ultrathin coatings have drawbacks, including incomplete shielding of the islet and low resistance against mechanical and biochemical stress. Moreover, long‐term stability in single‐layer conformal coatings is often insufficient, as it unwinds due to islet membrane turnover.^[^ ^174^ ^]^

#### 4.2.2 Layer‐by‐Layer (LBL) Encapsulation

In LBL encapsulation, individual islets or cells undergo the coating process with multiple alternating layers of positively and negatively charged natural and/or synthetic biocompatible polymers.^[^ ^164^ ^]^ This process typically begins with polycation deposition on the negatively charged islet surface.^[^ ^175^ ^]^ However, alternative approaches based on hydrogen or covalent bonds, or hydrophobic interactions have also been developed due to concerns about the cytotoxicity of polycations. LBL coating provides high flexibility in material selection and the number of assembled nano‐thin layers, as well as the option to localize certain agents, such as peptide sequences or drugs, within nanometers of the islet.^[^ ^158^ ^]^ Kim et al. introduced a novel approach for nanoencapsulation of NPIs using polymersomes based on poly(ethylene glycol‐block‐poly DL‐lactic acid) (PEG‐b‐PLA).^[^ ^176^ ^]^ When transplanted under the kidney capsule in mice, the nanoencapsulated NPIs exhibited prolonged immunoisolation and normal production and release of insulin.^[^ ^176^ ^]^ In a subsequent study, the same group used poly(ethylene glycol‐block‐poly lactide) for nanoencapsulation of NPIs, demonstrating more efficient immunoisolation and holding great promise for clinical application.^[^ ^177^ ^]^ Another study utilized LBL assembly to encapsulate mouse pancreatic β‐cell spheroids using fibronectin and gelatin, resulting in improved glucose sensitivity after transplantation into diabetic mice.^[^ ^178^ ^]^ LBL assembly employing tannic acid (TA) benefits from the antioxidative and immunomodulatory properties of this natural polyphenol.^[^ ^179^ ^]^ Barra et al. encapsulated NPIs in multiple layers of TA and poly‐N‐vinylpyrrolidone (PVPON).^[^ ^180^ ^]^ After transplantation into hyperglycemic mice, the TA‐PVPON encapsulated NPIs restored euglycemia and glucose tolerance at a level similar to non‐encapsulated NPIs. Moreover, they exhibited prolonged survival, reduced expression of proinflammatory genes, and a diminished innate immune response.^[^ ^180^ ^]^ In a separate study, non‐human primate islets were shielded with polyethylene glycol plus heparin using an LBL approach and transplanted into cynomolgus monkeys.^[^ ^181^ ^]^ In addition to demonstrating C‐peptide positive grafts, nanoencapsulated islets also reduced factors responsible for IBMIR in vitro.^[^ ^181^ ^]^ Additional details regarding functionalized ultrathin coatings with localized immunomodulatory properties are provided in Section 5 of the manuscript.

### 4.3 Macroencapsulation

**Figure 5:**
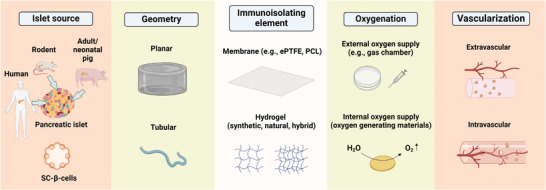
A broad perspective on feature diversity in islet macroencapsulation. Islets, derived from diverse sources such as humans, rodents, and both adult and neonatal pigs, contribute to this diversity. The encapsulation of islets involves the use of devices varying in shapes and sizes, crafted from a variety of immunoisolating biocompatible materials. Macroencapsulation devices, equipped with either external or internal oxygen sources, serve to prevent post‐transplantation hypoxia. Exploiting the adaptable design of macroencapsulation devices, both extravascular and intravascular models have been created and subjected to testing. This versatility in design offers a platform for exploring innovative approaches to islet encapsulation, enhancing the potential for successful transplantation outcomes (created with BioRender.com).

In contrast to nano‐ and microencapsulation of islets, macroencapsulation approaches have been developed to ensure a definitive localization and easy retrieval of the total transplanted islet mass in the event of graft failure.^[^ ^182^ ^]^ Due to the relatively large size of macroencapsulation devices, only one or very few of them are needed to transplant a sufficient number of islets to ensure the desired therapeutic outcome.^[^ ^183^ ^]^ Moreover, the larger dimensions (>1 mm in at least one direction) of macroencapsulation devices allow for flexibility in geometry, design, and complexity. Moreover, a combination of different customized materials can be used to achieve multifunctionality (**Figure** **5**). For instance, incorporating oxygen supply modules in macroencapsulation devices to prevent hypoxia, especially in the early post‐transplantation period, is beneficial for the viability and functionality of the encapsulated islets.

Macroencapsulation devices can be categorized into intravascular and extravascular types (Figure 5). Intravascular devices consist of hollow fibers or tubes made of a semipermeable membrane and are connected to the recipient's vascular system, ensuring an efficient exchange of materials.^[^ ^184^ ^]^ Despite many successful animal studies, intravascular devices are not the preferred version of macroencapsulation due to the high risk of blood clotting and infection, as well as the requirement for a complex surgical procedure for their implantation.^[^ ^184^ ^]^ In contrast, extravascular macroencapsulation devices are implanted in extrahepatic sites, such as the abdominal cavity, under the skin, or in the omentum, without an intravascular shunt, and can be easily removed.^[^ ^185^ ^]^ The major drawback of extravascular macroencapsulation devices is often insufficient vascularization, leading to reduced exchange and diffusion rates of nutrients and oxygen. This can result in hypoxia, gradual ischemia, and ultimately failure of the encapsulated islets.^[^ ^186^ ^]^

Macroencapsulation devices have been created using a wide range of natural, semi‐synthetic, or synthetic materials.^[^ ^187^ ^]^ In pure hydrogel‐based macroencapsulation approaches, the hydrogel serves both as a new artificial islet microenvironment to secure the islets and support their survival and function and as a permselective element to protect the islets from the host´s immune system, similar to most microencapsulation approaches.^[^ ^140^ ^]^ For instance, planar macroencapsulation devices feature a central layer containing islets embedded in materials such as alginate^[^ ^188^ ^]^ or collagen,^[^ ^189^ ^]^ covered by an acellular alginate layer on each side, thus forming a uniform immunoprotective barrier. Following subcutaneous transplantation into diabetic monkeys, the latter device reversed hyperglycemia for up to 6 months without immunosuppression.^[^ ^189^ ^]^ However, such macroencapsulation devices based solely on hydrogels often lack mechanical stability. This challenge can be effectively tackled by incorporating reinforcing elements like polymer meshes, fibers, scaffolds, or capsules into hydrogel‐based macrodevices. Alternatively, macroencapsulation devices using polymer membranes as (additional) permselective elements for immunoisolation, such as polytetrafluoroethylene (PTFE),^[^ ^190^ ^]^ cellulose acetate,^[^ ^191^ ^]^ or poly(ether sulfone)/poly(N‐vinylpyrrolidone) (PES/PVP) blends,^[^ ^192^ ^]^ have been designed to increase robustness.

The TheraCyte device, developed by Baxter Healthcare in the 1990s, is one of the most studied macroencapsulation devices, featuring an inner PTFE membrane with a pore size of 0.4 µm to maintain immunoisolation and an outer PTFE membrane with a pore size of 5 µm designed to promote vascularization around the graft.^[^ ^190a^ ^]^ Simultaneously, it contains a polyester mesh on its outermost surface to ensure mechanical stability and maintain its planar shape in vivo.^[^ ^190a^ ^]^ The TheraCyte device has shown promising results in various transplantation studies involving different animal models. In experiments with rats, allogeneic islets placed in the TheraCyte device and transplanted under the kidney capsule maintained their function for 6 months in both immunized and nonimmunized recipients, demonstrating the potential longevity of islet function within the device.^[^ ^193^ ^]^ Additionally, when the TheraCyte device filled with NPIs was subcutaneously transplanted into diabetic mice, it successfully reversed hyperglycemia for 16 weeks, indicating its efficacy in glycemic control.^[^ ^194^ ^]^ Further studies by Elliott et al. involved subcutaneous transplantation of the TheraCyte device containing NPIs into non‐diabetic cynomolgus monkeys, demonstrating effective immunoprotection of the encapsulated islets for 8 weeks.^[^ ^194^ ^]^ Notably, the recipients did not show any evidence of infection with porcine endogenous retroviruses or other endemic pig viruses.^[^ ^194^ ^]^ However, the long‐term protection provided by the TheraCyte device was limited potentially due to factors such as the relatively large pore size of the membrane and lack of sufficient oxygen supply to the encapsulated islets due to larger diffusion distance.

The development and evolution of ViaCyte Inc.’s encapsulation devices for the transplantation of insulin‐secreting cells (pancreatic endoderm cells or PECs) represent significant advancements in the field of diabetes treatment. In 2014, ViaCyte Inc. conducted the first‐in‐human phase I/II clinical trial using the Encaptra device (PEC‐Encap or VC‐01), which housed human embryonic stem cell‐derived insulin‐secreting cells (pancreatic endoderm cells (PECs), PEC‐01), developed using TheraCyte technology upon patent expiry.^[^ ^190^, ^195^ ^]^ This rectangular‐shaped device demonstrated immunoprotection, with encapsulated cells producing insulin in patients with T1DM. However, the device did not promote sufficient vascularization of the graft, and the viability and function of the cells were significantly reduced post‐implantation.^[^ ^190^, ^196^ ^]^ In 2017, ViaCyte designed a modified device (VC‐02 or PEC‐Direct) with multiple large across‐membrane pores to promote cross‐membrane vascularization.^[^ ^197^ ^]^ Clinical trials using VC‐02 showed promising outcomes, with an increase in fasting C‐peptide levels and a 20% reduction in exogenous insulin requirement during the 1 year follow‐up period.^[^ ^197^, ^198^ ^]^ However, this “open” device did not provide sufficient immunoisolation, necessitating systemic immunosuppression of the recipients.^[^ ^197^, ^198^ ^]^ To address the need for immunoisolation without systemic immunosuppression, ViaCyte further modified its PEC‐Encap device using an expanded PTFE (ePTFE) membrane with both immuno‐isolatory and pro‐angiogenic properties.^[^ ^198^ ^]^ Using this modified version, ViaCyte commenced a clinical trial without systemic immunosuppression of the recipients, however, the results are not yet available.^[^ ^199^ ^]^ Recently, ViaCyte partnered with CRISPR Therapeutics to develop a further upgraded device called VCTX210 or PEC‐QT. This device contained PECs from genetically engineered stem cell clones, lacking β2M and expressing PD‐L1 and HLA‐E transgenes to minimize the effect of the host immune system on the graft.^[^ ^198^, ^200^ ^]^ A new clinical trial using VCTX210 was commenced in 2022, although the results have not been published yet.^[^ ^201^ ^]^ In 2022, Vertex Pharmaceuticals acquired Viacyte to expand its T1DM islet cell therapy pipeline. Vertex's allogeneic stem cell‐based product (VX‐880) led to insulin independence in 2 T1DM patients during a 1‐year follow‐up in clinical trials.^[^ ^202^ ^]^ However, in January 2024 Vertex announced a pause in the VX‐880 T1DM human trial after two unrelated participant deaths. Despite this setback, Vertex reassured that the clinical trial for VX‐264, which encapsulates the same VX‐880 cells in a device designed to eliminate the need for chronic immunosuppressants, would proceed unaffected by the VX‐880 pause.^[^ ^203^ ^]^

The β‐Air device and the concept of using oxygen‐generating materials represent innovative approaches in the field of macroencapsulation for islet transplantation. In this disk‐shaped device, the islets are immunoisolated by a composite membrane containing two hydrophilic ePTFE membranes (25 µm thick and 0.45 µm pore size) impregnated with high mannuronic acid alginate.^[^ ^204^ ^]^ The device is designed as an extravascular system, meaning it is implanted in extrahepatic sites without a direct connection to the recipient's vascular system. It contains a compartment housing islets embedded in an alginate hydrogel, and adjacent to it, there is a user‐refillable oxygen compartment. The oxygen compartment addresses the challenge of lack of sufficient oxygen supply to the encapsulated islets, a crucial factor to their viability and function. Ludwig et al. assessed the feasibility of the β‐Air device in xenotransplantation of NPIs into diabetic rhesus macaques.^[^ ^204^ ^]^ They reported a reduction in the insulin requirement of the host without any viral transmission, indicating the potential effectiveness of the β‐Air macroencapsulation device.^[^ ^204^ ^]^ Alternatively, adequate oxygenation of islets in macroencapsulation devices might be achieved by oxygen‐generating materials. Compounds of this nature generate oxygen through the hydrolysis of inorganic peroxide particles embedded in a matrix. In a notable example, Coronel et al. created OxySite by utilizing poly(dimethylsiloxane) (PDMS) as a matrix to embed calcium peroxide particles. This innovative system demonstrated the capacity to generate oxygen for more than 1 month. Moreover, it significantly enhanced the survival of cells encapsulated in a hydrogel surrounding the PDMS scaffold, particularly under hypoxic conditions.^[^ ^205^ ^]^

In addition to so far discussed planar geometries and cylindrical macrocapsule designs, there have been reports on tubular macroencapsulation devices. The utilization of a tubular geometry is anticipated to facilitate higher mass transfer across the interface between the device and host due to its high surface area‐to‐volume ratio. A recent example is the Thread‐Reinforced Alginate Fiber For Islet Encapsulation (TRAFFIC) device described by An et al.^[^ ^206^ ^]^ This device was produced from nylon sutures coated with nanoporous poly(methyl methacrylate) (PMMA), which releases Ca^2+^. The modified suture was then coated with rat islets suspended in an alginate solution. The authors demonstrated the mechanical robustness and durable functionality of the TRAFFIC device in diabetic mice. Another innovative concept for strengthening tubular hydrogel structures in macroencapsulation has been explored by Wang et al.^[^ ^207^ ^]^ They developed the Nanofiber Integrated Cell Encapsulation (NICE) device, consisting of a highly porous and durable nanofibrous skin created through electrospinning a biocompatible medical‐grade thermoplastic silicone‐polycarbonate‐urethane, along with an immunoprotective alginate hydrogel core. The study revealed that stem cell‐derived β‐cells housed in the NICE device exhibited long‐term functionality after transplantation into the abdominal cavity of diabetic mice.

**Figure 6:**
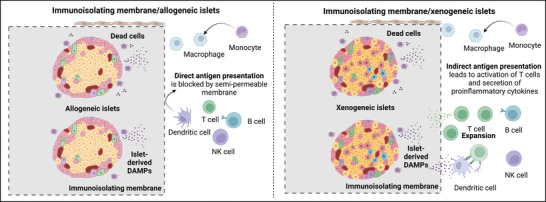
Immunoprotection of allogeneic and xenogeneic islets by immunoisolation (created with BioRender.com).

While the immunoisolating element, such as hydrogel or membrane, offers immunoprotection by blocking the direct recognition pathway, it often falls short of completely avoiding the immune response to the implanted graft, leading to fibrotic overgrowth around the encapsulated islets and potential graft failure.^[^ ^208^ ^]^ Notably, small molecules harmful to islets, such as chemokines/cytokines and nitric oxide, may pass through the capsule membrane and/or the hydrogel.^[^ ^209^ ^]^ Moreover, damaged islet cells within the capsules can release DAMPs, initiating early foreign body responses (FBRs) that trigger an immune response.^[^ ^184^ ^]^ DAMPs attract innate immune cells such as neutrophils, dendritic cells, and macrophages, leading to the production of proinflammatory cytokines. These infiltrating cells adhere to the outer layer of the immunoisolating material, obstructing the semipermeable membrane, blocking the diffusion of oxygen and nutrients, causing hypoxia, and ultimately resulting in the destruction of the encapsulated islets.^[^ ^184^ ^]^ Furthermore, host APCs presenting xeno‐epitopes can activate T cells, prompting the release of proinflammatory cytokines, which can cause xenograft rejection even in the absence of fibrotic overgrowth (**Figure** **6**).^[^ ^184^ ^]^ To tackle these challenges, various cell‐ and/or material‐based immunomodulation strategies have been developed. These strategies aim to alleviate the local inflammatory response and prevent fibrotic overgrowth on encapsulation devices. They can be applied in combination with immunoisolating approaches and also in macroscopic “open” (cell‐permissive) β‐cell replacement approaches without a permselective element, providing a means to protect the islets in the device from the host immune system. Certain strategies aimed at mitigating FBR in encapsulation approaches, such as material selection and the delivery of anti‐inflammatory agents, have been previously outlined in sections 4.1 and 4.2. In the upcoming Section 5, we will delve into partly biomaterial‐assisted approaches that involve providing immunomodulatory cells, agents, and/or cues to enable highly localized immunomodulation, offering an alternative or complementary method to immunoisolation and/or gene‐editing of the islet graft.

## 5 Accessory Cell‐ and/or Biomaterial‐assisted Local Immunomodulation Strategies

**Figure 7:**
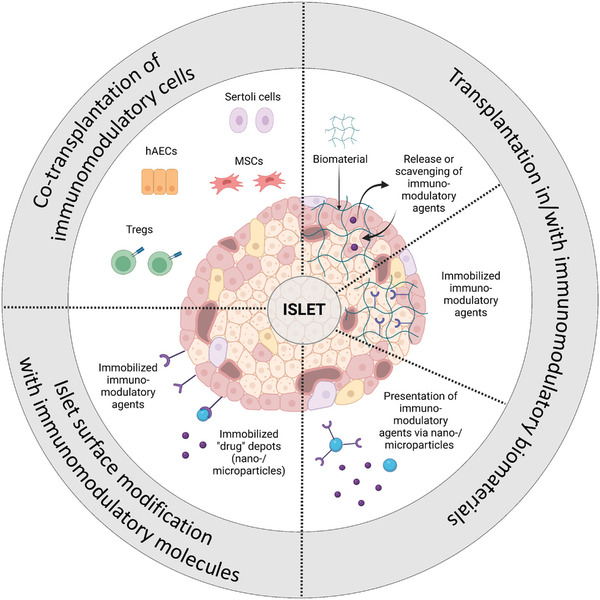
Immunoprotection by local immunomodulation through co‐transplantation of immunomodulatory cells, use of immunomodulatory biomaterials, and islet surface modifications (created with BioRender.com).

In the context of local immunomodulation, we focus on the strategies used to establish a local immunoprotective microenvironment for the graft through local presentation of instructional cues without generating a systemic effect in the recipient's body. These local cues can target specific immune pathways that are activated following the transplantation of either free or encapsulated islets. It is important to note that while the use of immunosuppressants or monoclonal antibodies is sometimes considered immunomodulation, they typically exert a systemic effect.^[^ ^26^ ^]^ Localized immunomodulation can generally be achieved by various methods: i) modifying the surface of islets/β‐cells by immobilizing immunomodulatory agents onto them; ii) releasing immunomodulatory agents from nano‐ or microcapsules that cover the islets/β‐cells into the local microenvironment; iii) utilizing biomaterials capable of presenting instructional cues, such as surface‐immobilized immunomodulatory agents, to the cells; iv) releasing or scavenging soluble agents into or from the local microenvironment (**Figure** **7**); v) co‐transplantation of the islets with accessory immunomodulatory cells (Figure 7); and vi) combinations thereof, which may involve simultaneous encapsulation or housing of the islets along with accessory cells in the biomaterial (reviewed in^[^ ^26^, ^184^, ^187^, ^210^ ^]^).

An example of direct surface modification of the islet graft is the biotinylation of the islet membrane to conjugate it with the tolerogenic molecule streptavidin‐Fas‐ligand (SA‐FasL) protein. FasL interacts with Fas (CD95) on T cells, triggering programmed cell death.^[^ ^211^ ^]^ Following transplantation in diabetic mice, SA‐FasL‐engineered allogeneic islets exhibited robust localized immune tolerance, effectively reversing hyperglycemia.^[^ ^212^ ^]^ Similarly, streptavidin‐CD47‐engineered allogeneic rat islets, following intraportal transplantation, mitigated IBMIR and enhanced islet engraftment compared to the control.^[^ ^78^ ^]^ The immunomodulatory agents can also be attached to the surface of biomaterials.^[^ ^26^ ^]^ For instance, FasL‐conjugated PEG microgels^[^ ^213^ ^]^ or FasL‐conjugated poly(lactide‐co‐glycolide) (PLG) scaffolds^[^ ^214^ ^]^ prolonged the survival of allogeneic islets and restored normoglycemia in mice without requiring chronic immunosuppression. Coronel et al. engineered biotinylated PEG microgels tethered with streptavidin/PD‐L1 (SA‐PD‐L1) for biomaterial‐assisted immunomodulation.^[^ ^215^ ^]^ Islets transplanted together with SA‐PD‐L1 microgels and sealed with an in situ cross‐linked PEG hydrogel containing VEGF exhibited a mean survival time of up to 50 days in nonautoimmune diabetic mice under a 15‐day low‐dose course of rapamycin, compared to 19 days in the rapamycin‐only group and 13 days in the control group without SA‐PD‐L1 microgels and rapamycin.^[^ ^215^ ^]^ More recently, the efficacy of this approach was tested in autoimmune‐prone NOD mice, demonstrating prolonged survival and function of syngeneic islets for over 60 days.^[^ ^216^ ^]^

Nguyen et al. utilized the approach of immobilizing nano‐/microparticles, releasing soluble immunomodulatory agents (drug depots), on the islet surface.^[^ ^217^ ^]^ They used a simple approach of engineering “cell‐particle hybrids” of pancreatic islets and bio‐inspired polydopamine‐coated poly(lactide‐co‐glycolide) (PLGA) microspheres for local sustained release of extreme low‐dose immunomodulatory molecules at the transplantation site.^[^ ^217^ ^]^ Alternatively, biomaterials (particles or open/closed scaffolds) releasing soluble immunomodulatory agents can be co‐transplanted with the islet graft. The impact of locally delivered PLGA microspheres releasing the immunosuppressant tacrolimus (FK506) and PEG‐based islet surface modification on xenogeneic islet survival was investigated in a mouse model.^[^ ^218^ ^]^ While PEGylation of islets alone was not enough to protect islets from early rejection, combined treatment with FK506‐releasing microspheres prolonged xenograft survival.^[^ ^218^ ^]^ In another study, Liu et al. utilized a microporous scaffold manufactured from poly(lactic‐co‐glycolic) acid (PLGA) loaded with transforming growth factor‐beta 1 (TGF‐β1) to mitigate the immune response and inflammation associated with transplanted islets.^[^ ^219^ ^]^ The release of TGF‐β1 from the scaffold resulted in a reduction in proinflammatory cytokines and leukocyte infiltration. This, in turn, facilitated the restoration of normoglycemia and prevented graft rejection. However, the effectiveness of this approach was observed to be limited to a duration of only 28 days post‐transplantation.^[^ ^219^ ^]^ In yet another strategy, the release of dexamethasone from a 3D macroporous scaffold based on PDMS significantly increased the engraftment of allogeneic islets in diabetic mice. Furthermore, this approach promoted the polarization of macrophages toward an anti‐inflammatory phenotype, illustrating its potential to alleviate the host immune response to transplanted islets.^[^ ^220^ ^]^ Biomaterial platforms can be also utilized to remove certain agents from the graft microenvironment. For instance, Lin et al. developed a peptide‐functionalized cytokine antagonizing PEG hydrogel (PEG‐peptide), capable of modulating local inflammation.^[^ ^221^ ^]^ These hydrogels exhibited efficient sequestration and neutralization of the pro‐inflammatory cytokine TNF‐α in vitro, as well as the modulation of local inflammation in vivo, thereby showcasing their potential for applications in drug delivery and tissue engineering.^[^ ^221^ ^]^ Similarly, Llacua et al. demonstrated that the incorporation of specific ECM molecules, such as collagen type IV along with RGD and PDSGR laminin sequences, into alginate microcapsules can effectively protect immunoisolated islets against cytokine toxicity.^[^ ^222^ ^]^

Various immunomodulatory cells have been explored for co‐transplantation with islets, including regulatory T cells (Tregs), mesenchymal stem cells (MSCs), dendritic cells, amniotic epithelial cells, or Sertoli cells, all demonstrating positive effects on islet survival (reviewed in^[^ ^26^, ^210^ ^]^). MSCs, in particular, have been extensively studied in islet transplantation due to their ability to create an immunosuppressive environment through the production of anti‐inflammatory cytokines and other regulatory molecules.^[^ ^26^ ^]^ Ren et al. conducted a study where adipose tissue‐derived MSCs were co‐transplanted with allogeneic mouse islets under the renal capsule of diabetic mice, resulting in increased islet revascularization and reduced inflammation.^[^ ^223^ ^]^ Ding et al. reported that allogeneic mouse islets co‐transplanted with MSCs exhibited a prolonged survival, lasting up to 95 days compared to 30 days in the control group, effectively reversing hyperglycemia in diabetic mice.^[^ ^224^ ^]^ Similarly, Li et al. demonstrated that co‐transplantation of allogeneic mouse islets with MSCs exhibited survival for up to 30 days compared to 16 days in the control group. This effect was attributed to the suppression of T‐cell response and cytokine secretion, leading to the reversal of hyperglycemia in diabetic mice.^[^ ^225^ ^]^

Innovative approaches have combined MSC co‐transplantation with subtherapeutic doses of immunosuppressive agents. Kim et al. utilized a combination of MSCs co‐transplantation and a subtherapeutic dose of cyclosporin A (CsA), extending the survival of allogeneic rat islets to 100 days.^[^ ^226^ ^]^ More recently, Wang et al. utilized engineered MSCs (eMSCs) expressing PD‐L1 and CTLA4‐Ig transgenes.^[^ ^227^ ^]^ The co‐transplantation of syngeneic or allogeneic islets with eMSCs in diabetic mice prolonged the islet survival up to 100 days without the need for systemic immunosuppression. In comparison, the islets transplanted with the non‐engineered MSCs exhibited a survival period of 20 days, while the islet‐only group had a shorter survival time of 14 days. Moreover, the islets co‐transplanted with eMSCs maintained normoglycemia for the entire 100‐day duration.^[^ ^227^ ^]^ Lebreton et al. took a unique approach by engineering insulin‐producing organoids from dissociated rat islet cells (ICs) and human amniotic epithelial cells (hAECs), transplanting them under the kidney capsule of diabetic mice.^[^ ^228^ ^]^ The IC‐hAEC organoids exhibited prolonged survival, improved graft revascularization, efficient maintenance of hyperglycemia, and higher C‐peptide levels compared to IC spheroid controls.^[^ ^228^ ^]^

Biomaterial‐assisted transplantation strategies either used open porous particles/scaffolds to improve co‐localization of islets and accessory cells or closed particles/scaffolds (encapsulation) to provide additional immunoisolation. Taking a comprehensive approach, Razavi et al. implemented a combination of islet encapsulation and co‐transplantation in their study.^[^ ^229^ ^]^ They first cultured mouse islets with MSCs to form an MSCs coating on the islets, followed by the conformal coating of the islets with alginate. When allotransplanted into mice, the double‐coated islets exhibited enhanced viability and maintained glycemic control throughout the 30‐day study period.^[^ ^229^ ^]^ Laporte et al. demonstrated that supplementing alginate microcapsules with both MSCs and RGD motifs is beneficial for human pancreatic islets viability and functionality compared to encapsulation using conventional alginate.^[^ ^230^ ^]^ In another study, MSCs and β‐cells were co‐cultured in a temperature‐responsive culture dish to obtain a cell sheet that was subsequently macroencapsulated in various alginate hydrogels to protect the cell sheets from immune attacks after transplantation.^[^ ^231^ ^]^ Survival and insulin secretion potential of the 3D macroencapsulated tissue was more successful than the transplantation of individual islet capsules.^[^ ^231^ ^]^ In a similar approach, Borg et al. designed open macroporous star‐shaped PEG‐heparin cryogel (starPEG‐heparin) scaffolds for the co‐culture of pancreatic islets and MSCs.^[^ ^232^ ^]^ They validated the insulin‐secreting function of mouse islets co‐cultured with MSCs in starPEG‐heparin scaffolds in vitro. Additionally, the in vivo feasibility of these scaffolds was assessed through subcutaneous transplantation into mice over a 7‐day period. After the recovery process, the scaffolds revealed the presence of both intact islets and MSCs.^[^ ^232^ ^]^ Graham et al. explored the co‐transplantation of allogeneic mouse islets and Tregs housed in poly(lactide‐co‐glycolide) (PLG) scaffolds at an extra‐hepatic and extra‐renal transplant site.^[^ ^233^ ^]^ This approach resulted in extended survival of the transplanted islets, including instances of indefinite protection, ultimately restoring normoglycemia in diabetic mice.^[^ ^233^ ^]^ Similarly, Valdes‐Gonzalez et al. reported the results of xenotransplants of neonatal porcine islets protected with porcine Sertoli cells inside an autologous collagen‐generating device to patients with T1DM transplanted subcutaneously in the anterior abdominal wall. They observed improved graft survival and function.^[^ ^234^ ^]^ Lastly, some approaches combine accessory cell‐ and biomaterial‐assisted local immunomodulation strategies. For instance, hybrid spheroids comprising MSCs with rapamycin (RAP)‐releasing PLGA microparticles were engineered to prevent immune rejection of islet xenografts in diabetic mice.^[^ ^235^ ^]^ Locoregional transplantation of these hybrid spheroids significantly prolonged islet survival times and promoted the generation of regional regulatory T cells.^[^ ^235^ ^]^ These collective findings underscore the potential efficacy of combining different immunomodulation strategies and immunoisolation to achieve prolonged islet graft survival and enhanced overall patient outcomes.

Citro et al. employed a decellularized organ to create a conducive microenvironment for islets and developed a functional bioengineered vascularized islet organ (VIO) ex vivo.^[^ ^236^ ^]^ Initially, they generated an acellular rat lung and repopulated acellular lung matrices with human umbilical vein endothelial cells (HUVECs). This process was undertaken to establish an intact hierarchical vascular tree within the lung matrix. The resulting structure then served as a scaffold for allogeneic islet engraftment before transplantation. The islets were successfully integrated into the bioengineered scaffold during a 7‐day culture period. The VIO demonstrated enhanced efficacy in reducing hyperglycemia in immune‐deficient NGS mice compared to fresh islets throughout the 30‐day assessment period.^[^ ^236^ ^]^ In a subsequent study, Citro et al. bioengineered a xenogeneic vascularized endocrine pancreas (VEP) using subject‐derived blood outgrowth endothelial cells (BOECs) and NPIs.^[^ ^237^ ^]^ This bioengineered VEP not only fostered neonatal islet maturation in vitro but also exhibited immediate in vivo functionality after transplantation, persisting for over 18 weeks in immune‐deficient NGS mice.^[^ ^237^ ^]^ While the in vivo experiments were conducted in immunodeficient mice and did not evaluate an immunoprotective effect, the VEP concept approach demonstrated successful integration of islets, improved efficacy in reducing hyperglycemia, and enhanced graft vascularization and function, showcasing potential advancements in islet transplantation techniques.^[^ ^236^, ^237^ ^]^

## 6 Conclusion and Perspective

Following successful clinical trials of islet transplantation, β‐cell replacement therapy has emerged as the ultimate therapeutic option for individuals with T1DM, aiming to restore physiologically regulated insulin secretion and achieve optimal metabolic control. Furthermore, to address the challenge of organ shortage significant advancement has been made toward xenotransplantation of porcine islets into humans. However, challenges like host immune responses and xenograft rejection persist. To address this, different strategies have been devised such as genetically modifying the donor pigs, employing immunoisolation through encapsulation, or utilizing local immunomodulation approaches, carrying the potential to prolong xenograft survival and function. Over the years, numerous studies have evaluated various genetic modifications, either individually or in combination, in donor pigs to evade the host immune response against xenograft. These genetic modifications include the elimination of 1 or more porcine xenoantigens and transgenic expression of human genes to counteract complement activation, inhibit coagulation dysregulation, mitigate innate and adaptive immune responses, and alleviate localized inflammation, all aimed at prolonging the function and survival of xenografts. While GM porcine islets have displayed promising outcomes in preclinical trials, discovering an optimal combination of genetic modifications exhibiting consistent successful results in preclinical studies remains an ongoing challenge. Nonetheless, once a donor pig line with an optimal genetic makeup is established, it can be easily propagated through breeding and serve as an unlimited source of islets for human xenotransplantation, presenting a significant advantage of this approach. Further exploration of the key immune pathways involved in islet graft rejection, using humanized mouse models and in vitro diabetes screening platforms utilizing human cells, will strengthen the development of more effective immunoprotection strategies.

In parallel, significant progress has been made in immunoisolation and local immunomodulation strategies as well. Immunoisolation involves physically isolating the encapsulated islets or cells from the recipient's immune system using a semipermeable membrane. This membrane allows for the passage of nutrients, oxygen, and insulin while blocking the entry of immune cells and antibodies, thus preventing rejection of the transplanted islets. Despite numerous preclinical and clinical trials that have proven the safety and feasibility of using encapsulated islets, challenges like diffusional limitations, immune‐mediated pericapsular growth and fibrosis, insufficient revascularization, biomaterial durability, long‐term graft survival, and achieving long‐term insulin independence persist. On the other hand, the immunomodulatory approaches refer to deliberate alteration of the local immune response within the microenvironment surrounding the encapsulated or free transplanted islets. Various immunomodulatory agents including synthetic drugs or anti‐inflammatory cytokines, or immunomodulatory cells including regulatory T cells and MSCs, capable of suppressing immune activation or inducing tolerance, have been assessed for their potential to diminish the likelihood of islet cell rejection. Primary approaches involve co‐transplanting islets with immunomodulatory cells, altering the surface of islets with immunomodulatory substances, and transplanting islets alongside or within immunomodulatory biomaterials. By targeting the transplantation site directly, local immunomodulation techniques can reduce the necessary dosage of immunomodulatory agents required to effectively shield the graft from the recipient's immune response. Moreover, immunomodulatory biomaterials not only function as reservoirs for releasing bioactive agents but can also sequester harmful elements from the transplant environment, such as proinflammatory cytokines, which might be helpful in reducing the infiltration of immune cells.

Recently, a combination of immunoisolation with immunomodulation strategies has been proposed to support the long‐term survival of the islet graft. Both immunoisolation and immunomodulation techniques aim to minimize the immune response against the transplanted cells, and their united effect may not only help increase graft survival but also reduce the requirement for systemic immunosuppression. The ideal scenario would be achieving local immunomodulation that effectively protects the graft from the recipient's immune system directly at the transplantation site, eliminating the need for systemic immunosuppression. The availability of a variety of biomaterials, the possibility to customize them for islet encapsulation, and the variety of available immunomodulation approaches are major advantages of these methodologies. Another key advantage of these approaches is their potential utility across various types of islets, including cadaveric allogeneic, syngeneic, and xenogeneic islets, as well as stem cell‐derived insulin‐producing β‐cells or organoids. Combining immunoisolation and immunomodulation strategies has shown promise in improving xenograft survival and function. However, the consistency of this approach is yet to be fully determined.

Several preclinical and clinical trials have fallen short of achieving sustained diabetes management criteria, specifically achieving insulin independence or significantly reducing insulin requirements beyond six months in NHPs, with clinically applicable immunosuppression regimens. Finding a combination of genetic modifications capable of countering various pathways involved in islet graft rejection, the utilization of innovative biomaterials to provide support for the encapsulated cells through an ECM‐mimicking matrix, or modulating the graft‐host interface by sequestering known detrimental factors holds the promise for enhancing the therapeutic outcome of islet xenotransplantation. As progress continues in refining β‐cell replacement therapy, collaborative efforts across disciplines hold the key to the success of islet xenotransplantation. A novel and encouraging strategy could involve merging genetic engineering, immunoisolation, and local immunomodulation. We speculate that co‐transplanting genetically modified porcine islets with immunomodulatory cells within an encapsulation device could synergize the immunoprotective effects of each approach. This approach not only has the potential to prolong the survival duration of the islets but could also alleviate the requirement for systemic immunosuppression therapy.

## Conflict of Interest

The authors declare no conflict of interest.
